# RepAHR: an improved approach for de novo repeat identification by assembly of the high-frequency reads

**DOI:** 10.1186/s12859-020-03779-w

**Published:** 2020-10-19

**Authors:** Xingyu Liao, Xin Gao, Xiankai Zhang, Fang-Xiang Wu, Jianxin Wang

**Affiliations:** 1 School of Computer Science and Engineering, Central South University, 932 South Lushan Rd, ChangSha, 410083 China; 2 Computational Bioscience Research Center, Computer, Electrical and Mathematical Sciences and Engineering Division, King Abdullah University of Science and Technology (KAUST), Thuwal, 23955 Saudi Arabia; 3 Biomedical Engineering and Department of Mechanical Engineering, University of Saskatchewan, Saskatoon, SKS7N5A9 Canada

**Keywords:** De novo repeat identification, NGS reads, The high-frequency *k-mers*, The high-frequency reads, Assembly

## Abstract

**Background:**

Repetitive sequences account for a large proportion of eukaryotes genomes. Identification of repetitive sequences plays a significant role in many applications, such as structural variation detection and genome assembly. Many existing de novo repeat identification pipelines or tools make use of assembly of the high-frequency *k-mers* to obtain repeats. However, a certain degree of sequence coverage is required for assemblers to get the desired assemblies. On the other hand, assemblers cut the reads into shorter *k-mers* for assembly, which may destroy the structure of the repetitive regions. For the above reasons, it is difficult to obtain complete and accurate repetitive regions in the genome by using existing tools.

**Results:**

In this study, we present a new method called RepAHR for de novo repeat identification by assembly of the high-frequency reads. Firstly, RepAHR scans next-generation sequencing (NGS) reads to find the high-frequency *k-mers*. Secondly, RepAHR filters the high-frequency reads from whole NGS reads according to certain rules based on the high-frequency *k-mer*. Finally, the high-frequency reads are assembled to generate repeats by using SPAdes, which is considered as an outstanding genome assembler with NGS sequences.

**Conlusions:**

We test RepAHR on five data sets, and the experimental results show that RepAHR outperforms RepARK and REPdenovo for detecting repeats in terms of N50, reference alignment ratio, coverage ratio of reference, mask ratio of Repbase and some other metrics.

## Background

The repetitive sequences are patterns of nucleic acids, which occur multiple times in genome with the same or approximate form. Based on their structure and distribution in the genome, repetitive sequences are classified into several types, i.e. tandem repeats, interspersed repeats and so on. Tandem repeats consists of repetitive elements adjacent to each other and they are categorized into satellites, minisatellites and microsatellites based on their repetitive element size and repetitive level. Interspersed repeats are dispersed throughout the genome and mainly composed of transposable elements (TEs). Transposable elements account for a large fraction of the genome and have influence on much of the mass of DNA in eukaryotic genomes [1]. In many organisms, repeated sequences make up a significant part of whole genomes, e.g. over two-thirds of the human genome [2], over 75% of the maize genome [3] and about 20% of the *Drosophila melanogaster* genome [4] are repetitive. For many basic analysis methods of genome sequences, such as de novo assembly, sequence alignment, sequence error correction, etc., repetitive sequences pose a challenge to these tasks [5].

In recent years, many methods have been proposed with the developments and applications of the high-throughput sequencing and single molecule sequencing. Tallymer [6] counts *k-mer* occurrences from sequence sets and puts *k-mer* into enhanced suffix arrays to find repetitive sequences. RepeatExplorer [7] is a collection of software tools with a web interface and utilizes a graph-based sequence clustering algorithm to facilitate de novo repeat identification. RepARK [8] creates repeat libraries from NGS reads by assembly of the high-frequency *k-mer* without using a reference genome, which has two working modes based on the assembly tools, RepARK Velvet and RepARK CLC. MixTaR [9] firstly detects raw tandem repeat patterns from short reads, then selects out long reads containing raw tandem repeat patterns, and finally regards the assembly of the short reads which overlap long reads as TR repeats. RepLong [10] creates a read overlap network and uses community detection algorithm to detect long repeats by using only Pacbio long reads. REPdenovo [11] counts *k-mer* frequency and assembles *k-mer* into raw contigs, then merges contigs from directed contig graph into scaffolds to obtain repeats. The *k-mer* frequency is typically calculated from the NGS reads and the repeats are obtained by assembly of the high frequency *k-mers*. For example, RepARK and REPdenovo are both methods based on this principle. However, these approaches have some drawbacks.

Firstly, the higher sequence coverage is a requirement for common assemblers to work properly. For example, EPGA [12] ,EPGA2 [13] and SPAdes [14] usually require read coverage of more than 30$\times$, in which SPAdes is considered as an outstanding genome assembler with NGS sequences. Compared with other sequence assemblers, SPAdes uses multiple *de Bruijn* graphs to construct contigs. This strategy can effectively reduce assembly errors while making full use of a variety of *k-mers* of different sizes to build more complete assemblies [15]. Due to the dual effects of sequencing bias and the complexity of the repetitive region structure, the fragment can be repeated from several times to serval thousand times. For *k-mers* converted from fragments with a large number of repetitions, their frequencies usually much higher than the average coverage of sequencing, and for others with few repetitions may not meet the basic coverage requirement. Therefore, the repetitive fragments with low repeatability are difficult to obtain by assembly, and the integrity of the test results is greatly affected.

Secondly, most of the widely-used NGS assemblers are designed based on the paired-end reads, which often resolve the branch path resulting from the repetitive region by using paired-end reads of large insertsizes. However, the length of the high frequency *k-mers* is too short compared with the reads, which is detrimental to the recovery of the repetitive segments. In addition, splitting reads into *k-mers* may destroy the structure of the repetitive regions, which means that assemblers do not perform well under such conditions.

Finally, due to the length of the *k-mer* is short, the sequencing error has a great influence on the result of the *k-mer* frequency counting. Assembly of *k-mers* may bring the sequencing errors within these *k-mers* into repeats, resulting the accuracy of the repetitive regions decreases. In this study, a method called RepAHR is proposed to overcome the shortcomings mentioned above and get more accurate repeats. Compared the repeats identified by RepAHR with these of RepARK and REPdenovo on five NGS datasets, the experimental results show that RepAHR outperforms RepARK Velvet, RepARK CLC and REPdenovo in some aspects.

## Results and discussion

### Metrics for evaluation

In order to comprehensively evaluate the performance of each tool, we use seventeen evaluation metrics in this experiment, which are Num, Max (bp), Min (bp), N50 (bp), N90 (bp), Avg_length (bp), AR (%), Alignment ratio (%), Multiple alignment ratio (%), Masked ratios on reference genome (%), Masked ratios on RepBase sequences (%), User Time (s), System Time (s), Percent of CPU this job got (%), Maximum resident set size (kbytes), Virtual Memory (kb) and File system outputs. Among them, ’Num’ denotes the number of segments in detection results, ’Max (kb)’ denotes the length of the largest segment in detection results, ’N50 (kb)’ is the length of the longest segment such that all the segments longer than this segment cover at least half (50%) of the total length of all segments, ’N90’ are calculated in a similar way, ’Avg_length (bp)’ denotes the average length of segments, ’AR (%)’ denotes alignment ratio of the high-frequency *k-mer* or the high frequency reads on RepBase sequences, ’Alignment ratio (%)’ is the proportion of fragments in the detected results that can be aligned to the reference genome, ’Multiple alignment ratio (%)’ is the proportion of fragments in the detection results that can be aligned to multiple locations on the reference genome, ’Masked ratios on reference genome (%)’ is the proportion of bases on the reference genome that can be covered by the detection results, ’Masked ratios on RepBase sequences (%)’ is the proportion of bases in RepBase that can be covered by the detection results, ’Virtual Memory (kb)’ indicates the peak virtual memory consumption of algorithms.

We evaluate the repeats identified by RepAHR, RepARK and REPdenovo on five NGS data sets. Table 1 shows the comparison of the repeats identified by RepAHR, RepARK and REPdenovo in terms of number of repeats, total bases number of repeats, N50 and length of repeats. REPdenovo can identify few repeats and its bases number is the least among the four methods, but the average length of its repeats is the longest. RepARK CLC identifies more repeats and bases number than REPdenovo, but repeats identified by RepARK CLC are shorter than repeats identified by REPdenovo. Compared with the other three tools, RepARK Velvet can identify more repeats, but the bases number of its repeats is less than RepAHR in *Drosophila melanogaster*, *Homo sapiens chr14* and *Mus musculus*. The repeats identified by RepARK Velvet have the shortest average length, which can be seen from the N50 and N90. RepAHR identifies fewer repeats than RepARK Velvet, but the size of repeats identified by RepAHR is larger than that obtained by RepARK Velvet. In terms of sequence length, the maximum repeat length identified by RepAHR is the longest of the four methods, but it can be seen from the N50 and N90 that the repeats identified by RepAHR also contains many short fragments. The long and short fragments combined together make the final repeats identified by RepAHR more complete.

**Table 1:** Metrics of repeats on five datasets

Species	Method	Num	Size (kbp)	Max/min	N50 (bp)	N90 (bp)
*Drosophila* *melanogaster*	RepARK CLC	818	518	6833/200	1040	255
	RepARK Velvet	4561	873	7587/57	285	87
	REPdenovo	52	61	8339/102	2843	397
	RepAHR	2647	1350	12,787/56	1350	98
*Saccharomyces* *cerevisiae*	RepARK CLC	545	291	8271/200	626	266
	RepARK Velvet	1457	394	9129/57	423	111
	REPdenovo	3	0.72	265/213	258	213
	RepAHR	392	219	9523/128	2089	183
*Acromyrmex* *echinatior*	RepARK CLC	485	249	8272/200	659	240
	RepARK Velvet	3931	559	5547/57	160	68
	REPdenovo	249	99	2143/100	597	182
	RepAHR	2699	514	10,701/88	285	86
$Homo \ sapiens$ *chr*14	RepARK CLC	105	29	594/201	273	216
	RepARK Velvet	846	106	574/57	140	80
	REPdenovo	14	9	5545/101	5545	211
	RepAHR	1738	219	2177/45	213	61
$Mus\ musculus$	RepARK CLC	3839	1835	17,062/200	565	236
	RepARK Velvet	47,232	2302	16,526/57	129	57
	REPdenovo	9376	12,652	14,827/100	3129	848
	RepAHR	77,891	19,201	28,893/150	503	222

$'Num'$ indicates the number of repeats. $'Size'$ indicates the total length of all repeats. $'Max'$ represents the length of the longest segment in the repeats. $'Min'$ represents the length of the shortest segment in the repeats. $'N50\, or\, N90'$ represents the length of the longest segment such that all the segments longer than this segment cover at least 50% or 90% of the total length of the assemblies

### The high-frequency *k-mers* and reads

Among RepAHR, RepARK Velvet, RepARK CLC and REPdenovo, the most obvious difference between RepAHR and the other three methods is that RepAHR does not directly use the high-frequency *k-mers* for sequence assembly. RepAHR first uses the high-frequency *k-mers* to find the high-frequency reads, then assembles the high-frequency reads to obtain repeats. In order to prove that the high-frequency reads have some advantages over the high-frequency *k-mers*, we compare the high-frequency *k-mers* and the high-frequency reads in experiments.

Repbase is the most widely-used database of repetitive DNA sequences in which the currently known repetitive DNA sequences of many eukaryotes are stored [16]. The similarity between a sequence and the sequences in Repbase can be used as a criterion for determining whether the sequence is a repetitive sequence. In the test, three NGS datasets (*D.mela*, *M.musc* and *H.sapi*) are used to obtain the high-frequency *k-mers*, and the high-frequency reads are selected by RepAHR from all reads according to the high-frequency *k-mers*. RepARK, REPdenovo and RepAHR differ in the method of determining the high-frequency *k-mers* threshold. In our test, the high-frequency *k-mers* is obtained using the high-frequency threshold of RepARK. The high-frequency *k-mers* and reads separated from three datasets (*D.mela*, *M.musc* and *H.sapi*) are aligned to the corresponding Repbase library for each species, respectively. The aligned sequences with overlaps are connected together to form a long segment called alignment segment.

**Fig. 1:**
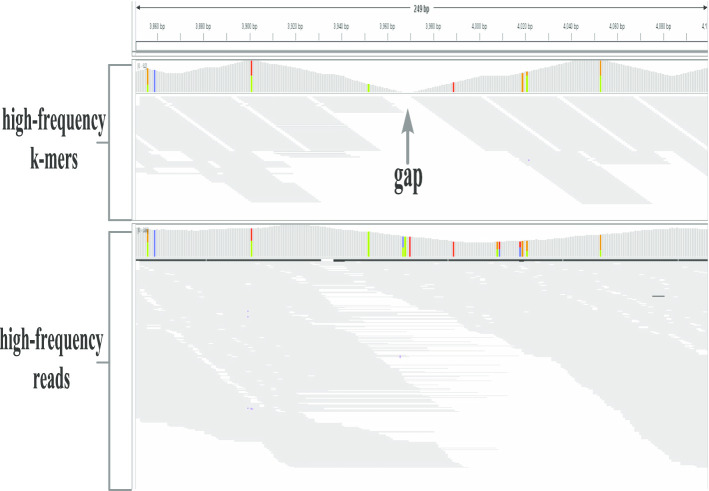
A visual example of the alignment of the high frequency *k-mers* and the high frequency reads with the segments in Repbase library

The comparison of the number, N50, the average length and the maximum length of aligned segments obtained based on the high-frequency k-mers and reads is shown in Table 2. N50 is the length of the longest contig such that all the contigs longer than this contig cover at least half of the genome being assembled [17]. In addition, the alignment ratios of the high-frequency *k-mers* and reads compared with the Repbase are shown in Table 2. In Table 2, the alignment ratios of the high-frequency reads are much higher than the alignment ratios of the high-frequency *k-mers*. It also can been seen that the number of aligned segments obtained by the high-frequency *k-mers* is much larger than that obtained by the high-frequency reads in three species. At the same time, the length of the alignment segments obtained by the high-frequency reads is greater than that obtained by the high-frequency *k-mers*, which can be seen from the variation of the N50 and the average length of detection results in Table 3. The maximum length of the aligned segments covered by the high-frequency reads is the same as the *k-mers*. This shows that the high-frequency reads and *k-mers* are similar in covering the long alignment segments. As can be seen from Table 3, the total number of alignment segments obtained by the high-frequency reads is less than that obtained by the high-frequency *k-mers*, but the number of alignment segments obtained by the high-frequency reads is greater than that obtained by the high-frequency *k-mers* in the long length portion. It can be inferred that the short alignment segments obtained by the high-frequency *k-mers* are formed by gaps in which continuous regions cannot be communicated. The visualization tool IGV [18] is used to demonstrate the alignment of the high-frequency reads and *k-mers* with Repbase sequences. Figure 1 shows the case where the sequence named BATUM_I in the *Drosophila melanogaster* Repbase library is covered by the high-frequency reads and *k-mers*. There is a gap on the region that is not covered by the high-frequency *k-mers*, while this region is completely covered by the high-frequency reads, which proves that the high-frequency reads is easier to obtain a complete repeat region.

**Table 2:** Metrics of alignment segments

Species	Segment type	Num	N50 (bp)	Avg length (bp)	Max (bp)	AR (%)
*Drosophila* *melanogaster*	High-frequency *k-mers*	4105	1291	187.36	8256	58.90
	High-frequency reads	1336	3336	542.81	8256	80.55
*Mus* *musculus*	High-frequency *k-mers*	2612	1142	262.49	7673	24.73
	High-frequency reads	421	5943	1203.55	7673	82.64
$Homo \ sapiens$ *chr*14	High-frequency *k-mers*	1481	239	112.05	6064	38.32
	High-frequency reads	242	394	338.63	6064	75.23

$'Num'$ indicates the number of alignment segments. $'N50'$ indicates the length of the longest segment such that all the segments longer than this segment cover at least 50% of the total length of all segments. $'Avg\,Length (bp)'$ indicates the average length of segments. $'Max'$ indicates the maximum length of a repeat. $'AR (\%)'$ indicates alignment ratio of the high-frequency *k-mers* or the high-frequency reads on Repbase sequences

**Table 3:** Length distribution of alignment segments

Species	Segment type	Total Num	> 1000 bp	> 2000 bp	> 4000 bp	> 6000 bp
*Drosophila* *melanogaster*	High-frequency *k-mers*	4105	113	78	52	14
	High-frequency reads	1336	139	87	60	19
*Mus* *musculus*	High-frequency *k-mers*	2612	93	58	41	30
	High-frequency reads	421	83	65	52	36
$Homo \ sapiens$ *chr*14	High-frequency *k-mers*	1481	5	3	2	0
	High-frequency reads	242	11	3	3	0

$'Total\, Num'$ indicates the number of alignment segments. $'$>$1000'$ indicates the number of alignment segments whose length is greater than 1000 bp, and the rest is the same

Based on the above results and analysis, it can be considered that the high-frequency reads is longer, more continuous, and more accurate than the high-frequency *k-mers*, and it is more suitable for obtaining repeats by sequence assembly. Compared with the previous two methods, RepAHR not only replaces the high-frequency *k-mers* with the high-frequency reads, but also preserves the information of the paired-end reads as much as possible to assist in the assembly of repetitive regions. It is well known that the paired-end reads can span hundreds to thousands of bp (base pair), so using its supporting information [19], RepAHR can assemble and identify longer repetitive regions.

### Verification of the authenticity of repeated sequences detected by RepAHR

**Fig. 2:**
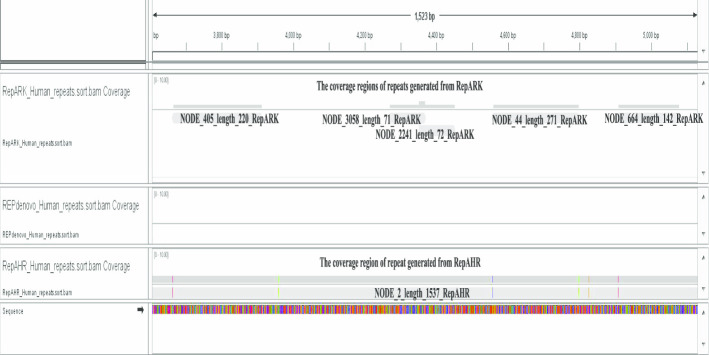
A special repetitive region on the human-r14 genome is covered by the detection results of each tools.

In order to prove that the repeats recognized by RepAHR are not only long , but also are the true repeats in the genome. We take a special repetitive region detected by three tools on dataset of human-r14 as an example to analyze, just as shown in Fig. 2. In Fig. 2, the first block shows the alignment relationships between the repetitive fragments detected by RepARK and the reference genome, the second block shows the alignment relationships between the repetitive fragments detected by REPdenovo and the reference genome, and the third block shows the alignment relationships between the repetitive fragments detected by RepAHR and the reference genome. From Fig. 2, we can find that the repetitive fragment detected by RepAHR is more complete than that obtained by RepARK, and there is no fragment detected by REPdenovo in this region. Therefore, RepAHR obtains the longest and the most complete repetitive fragment in this region. Next, we need to prove whether the repetitive fragment obtained by RepAHR is real. In order to achieve the purpose of verification, we collected the repeated fragments obtained by different tools in this region, and aligned them to the human-r14 reference sequence by using the bowtie2 [20] aligner. The alignment results show that the repetitive fragment detected by RepAHR can be aligned to different locations on the human-r14 reference sequence, and its coverage regions include the coverage regions of the RepARK’s detection results. The sequence fragment detected by RepAHR is not only a true repeat in the genome, but also the longest and most complete fragment in the detection results of three tools.

**Fig. 3:**
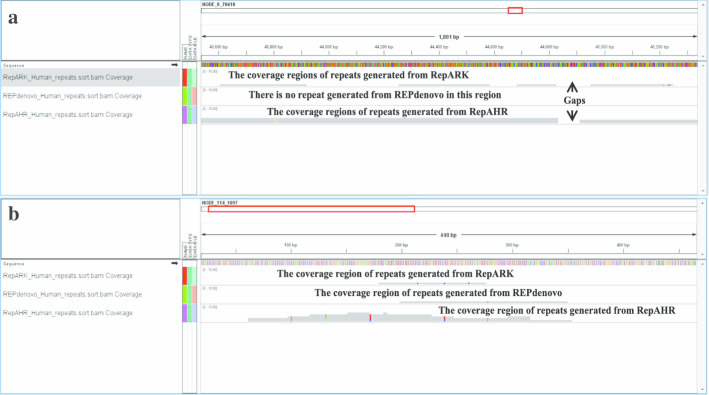
An practical example of the alignment of fragments obtained by different tools and the human-r14 reference sequence

In order to prove that the repetitive regions detected by RepAHR which cannot or cannot fully be identified by other tools, we compared the repetitive regions detected by the three tools on the human-r14 dataset. The benchmark for comparison is the repetitive regions on the human-r14 dataset provided by a third-party library, and the comparison results are shown in Fig. 3. From Fig. 3, we find that RepAHR can detect some specific regions that other tools cannot identify or cannot fully identify. In this step, Bowtie2 [20] is used as a aligner.

In order to verify the influence of read coverage on the detection effect of each tools, we designed a simulation experiment based on the human-r14 reference sequence. The specific process of the simulation experiment is as follows: Firstly, four sets of simulation sequencing libraries with different coverage are generated by ART (an NGS read simulator) using the human-r14 reference sequence as the template. Secondly, we have tested the three tools on four simulated sequencing libraries, and the test results are shown in Table 4 The average read coverage represents the average sequencing depth at each regions of the genome, which reflects the sequencing level of the entire genome. From Table 4, we can see that the average read coverage have a certain influence on the detection results of each tools. From the overall trend, the max length and N50 of the fragments in the test results increases as the average coverage of the reads increases. The main reason for this phenomenon is that the sequence assembly process is extremely susceptible to changes in read coverage. For example, Velvet, SOAPdenovo2, Abyss, IDBA and SPAdes usually require read coverage of more than 30X to meet the basic requirements of assembly, otherwise the assemblies will be very fragmented. From the experimental results shown in Table 4, we can find that the impact of read coverage on the detection results of tools is inevitable, and this impact is not specific to RepAHR, but also to these two similar tools.

**Table 4:** The influence of read coverage on the detection effect of each tools

Species	Method	Cov/read_len (bp)	Num	MA_ratio (%)	Max/min	N50 (bp)	N90 (bp)
$Homo \ sapiens$ *chr*14	RepAHR	30X/100	33	100.0	833/100	286	108
	RepAHR	60X/100	32	100.0	855/100	238	126
	RepAHR	90X/100	35	100.0	1087/100	238	116
	RepAHR	120X/100	30	100.0	2057/100	238	116
	REPdenovo	30X/100	13	100.0	5079/72	5079	173
	REPdenovo	60X/100	13	100.0	5079/72	5079	147
	REPdenovo	90X/100	9	100.0	5079/76	5079	198
	REPdenovo	120X/100	11	100.0	5078/79	5078	173
	RepARK Velvet	30X/100	419	99.82	382/29	99	0
	RepARK Velvet	60X/100	545	99.76	430/29	104	0
	RepARK Velvet	90X/100	569	99.82	430/29	102	0
	RepARK Velvet	120X/100	1992	99.85	1249/29	119	64

$'Cov/read\_len\, (bp)'$ indicates the average coverage of sequencing and the average length of reads. $'Num'$ indicates the number of repeats. $'MA\_ratio\, (\%)'$ represents the multiple alignment ratio of repeats. $'Max'$ represents the length of the longest segment in the repeats. $'Min'$ represents the length of the shortest segment in detected repetitive segments. $'N50\,or\,N90'$ represents the length of the longest segment such that all the segments longer than this segment cover at least 50% or 90% of the total length of the assemblies

Generally, there is a certain conversion relationship (just as shown in equation (2) of the main text) between the average coverage of reads and the frequency of *k-mers* converted from these reads. Therefore, we can obtain the average read coverage by using the *k-mer* frequency distribution information, and we can also obtain the high-frequency *k-mers* and the high-frequency reads in the global genome based on the average read coverage. From a global perspective, assuming that the sequencing is roughly balanced, the high-frequency *k-mers* must be from the repetitive regions. Therefore, the reads containing more the high-frequency *k-mers* must also from the repetitive regions. The conclusion is that as long as the high-frequency *k-mers* contained in a read reaches a certain proportion, this read must be included in RepAHR without being missed. On the contrary, the reads missed by RepAHR must be those that do not meet the above requirements. We are not sure whether they come from the repetitive regions.

The running time, CPU and memory consumption of RepAHR and other tools on five datasets are shown in Table 5. From the perspective of running time, RepARK has the most advantages among these three tools, and the longest running tool is sometimes REPdenovo and sometimes RepAHR. From the perspective of memory consumption, RepAHR and RepARK are more dominant on some datasets, for example, the memory consumption of the two tools on the first three datasets is almost the same. From the perspective of CPU usage, REPdenovo has the highest degree of parallelism. From the perspective of system throughout, RepAHR is dominant in most cases.

**Table 5:** Statistics of running time and memory consumption of each tools on five datasets

Species	Method	User time (s)	System time (s)	Percent of CPU this job got (%)	Maximum resident set size (kbytes)	File system outputs
*Drosophila* *melanogaster*	RepARK CLC	1462.63	57.80	101	9,519,212	53,956,504
	RepARK Velvet	1537.49	55.53	101	9,519,228	54,155,904
	REPdenovo	4643.07	44.52	1211	13,194,748	42,044,664
	RepAHR	4735.17	125.72	868	9,530,504	60,185,232
*Saccharomyces* *cerevisiae*	RepARK CLC	792.39	25.27	101	4,875,512	29,969,488
	RepARK Velvet	943.62	27.16	100	4,875,512	29,878,280
	REPdenovo	11,229.97	5803.70	316	59,960,388	74,127,816
	RepAHR	2507.21	102.31	856	4,876,644	34,047,800
*Acromyrmex* *echinatior*	RepARK CLC	3437.92	154.23	96	18,693,376	86,491,600
	RepARK Velvet	3929.52	194.82	100	18,693,376	86,459,800
	REPdenovo	17,487.81	112.37	1272	25,530,952	77,131,304
	RepAHR	20,763.21	288.73	1554	18,694,492	97,486,232
$Homo \ sapiens$ *chr*14	RepARK CLC	1224.70	48.64	102	4,875,512	43,523,184
	RepARK Velvet	1341.06	42.27	101	4,875,532	43,620,064
	REPdenovo	5482.66	40.12	1280	5,853,720	37,027,688
	RepAHR	4273.05	173.32	771	26,354,272	47,734,280
$Mus\ musculus$	RepARK CLC	9245.10	709.18	101	36,635,684	329,177,184
	RepARK Velvet	8977.43	421.34	100	36,635,676	333,214,224
	REPdenovo	52,429.50	1047.07	1178	133,069,768	312,131,728
	RepAHR	633,171.42	8987.20	2701	257,398,252	598,113,592

$'User \, Time (s)'$ indicates the process spent in user mode. $'System \, Time (s)'$ indicates the process spent in system mode. $'Percent\,of\,CPU\,this\,job\,got'$ represents the percentage of the job using CPU. $'Maximum\, resident\, set\, size'$ represents the maximum resident memory size of the job. $'File\, system\, outputs'$ indicates the number of files output by this job

### Repeats on reference genomes

In the experiment, Bowtie2 [20] is used as a aligner to obtain the overall and the multiple alignment ratios between the repeats and their respective reference sequences on the five data sets. The overall alignment ratio refers to the ratio of the number of repeats that can be aligned to the reference genome at least one position to the total number of repeats. The multiple alignment ratio refers to the ratio of the number of repeats that can be aligned to the reference genome multiple positions to the total number of repeats.

**Fig. 4:**
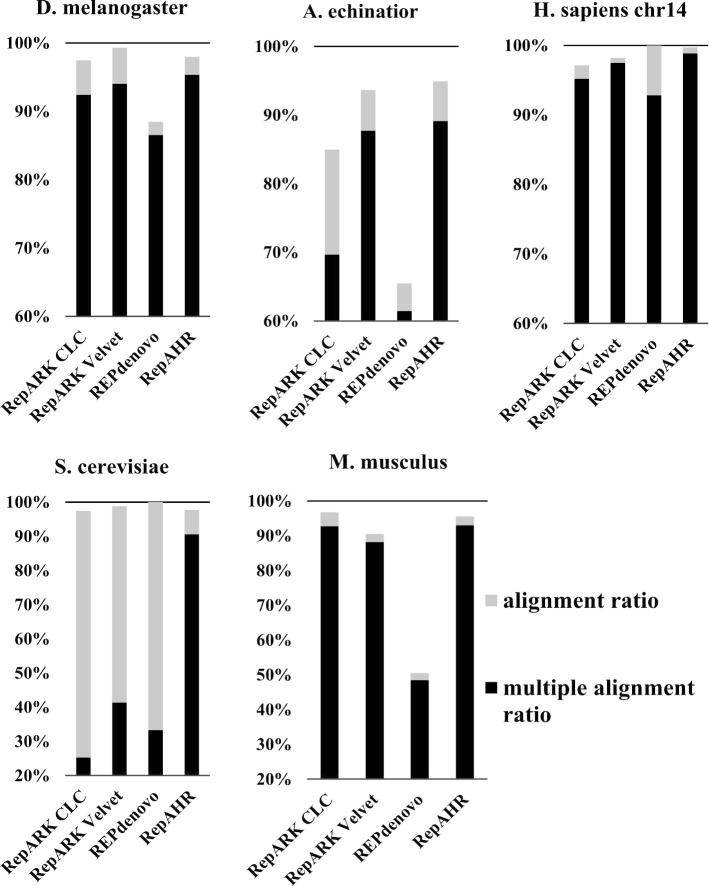
Alignment ratios and multiple alignment ratios of repeats which generated from four tools on five different datasets. ‘Alignment ratios(%)’ is the proportion of fragments in the detected results that can be aligned to the reference genome, and ‘Multiple alignment ratios(%)’ is the proportion of fragments in the detected results that can be aligned to multiple locations on the reference genome

**Fig. 5:**
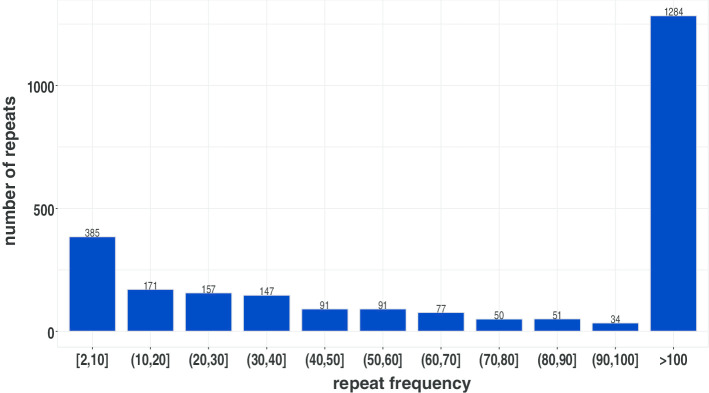
The frequency distribution of segments in detected results generated from RepAHR on dataset of Drosophila melanogaster

As can be seen from Table 6 and Fig. 4, RepAHR gets the higher multiple alignment ratios than the other three methods on the five data sets. In particular, RepAHR has obvious advantages in the multiple alignment ratio on *Saccharomyces cerevisiae* dataset. The experimental results show that the repeats identified by RepAHR are more repetitive and accurate than the other three methods. As can be seen from Table 6, the proportion of repeats identified by RepAHR can be aligned with multiple positions of the reference genome is higher than the other tools. For example, Bowtie2 is used to align repeats identified by RepAHR to the reference genome of *D. melanogaster*. The alignment results show that more than half of these repeats can be aligned to more than 100 positions of the reference genome, just as shown in Fig. 5.

**Table 6:** Alignment ratios and multiple alignment ratios of repeats on five data sets

Species	Method	Num	Alignment ratio (%)	Multiple alignment ratio (%)
*Drosophila* *melanogaster*	RepARK CLC	818	97.43	92.42
	RepARK Velvet	4561	99.28	94.04
	REPdenovo	52	88.46	86.54
	RepAHR	2647	97.96	95.35
*Saccharomyces* *cerevisiae*	RepARK CLC	545	97.42	25.32
	RepARK Velvet	1457	98.76	41.39
	REPdenovo	3	100.0	33.33
	RepAHR	392	97.70	90.56
*Acromyrmex* *echinatior*	RepARK CLC	485	84.95	69.69
	RepARK Velvet	3931	93.64	87.75
	REPdenovo	249	65.46	61.45
	RepAHR	2699	94.89	89.14
$Homo \ sapiens$ *chr*14	RepARK CLC	105	97.14	95.24
	RepARK Velvet	846	98.23	97.52
	REPdenovo	14	100.0	100.0
	RepAHR	1738	99.71	98.85
$Mus\ musculus$	RepARK CLC	3839	96.77	92.81
	RepARK Velvet	47,232	90.47	88.20
	REPdenovo	9376	50.53	48.54
	RepAHR	77,891	95.56	93.05

$'alignment\ \ ratio'$ indicates the ratio of the number of repeats aligned to the reference genome at least one position to the total number of repeats. $'multiple\ \ alignment\ \ ratio'$ indicates the ratio of the number of repeats aligned to the reference genome multiple positions to the total number of repeats

**Fig. 6:**
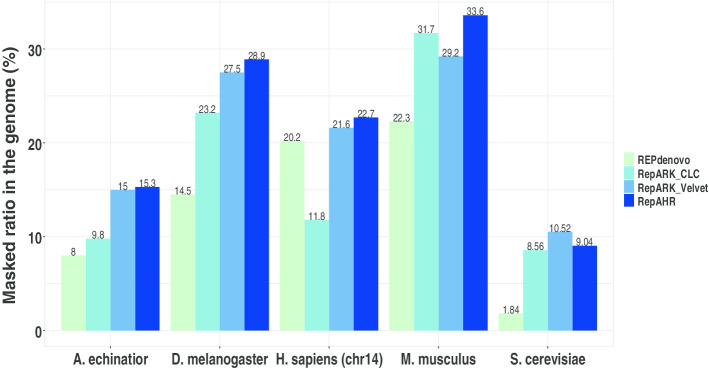
Masked ratios on reference genome. 'Masked ratios on reference genome(%)' is the proportion of bases on the reference genome marked as the repeats generated from the four tools, which is measured by RepeatMasker

RepeatMasker [21] is a tool for masking interspersed repeats and simple tandem repeats by using sequence alignment, which is used to obtain the masked ratio of the repeats identified by RepAHR, RepARK CLC, RepARK Velvet and REPdenvo on the reference, respectively. As can be seen from Fig. 6, the masking ratio of the repetitive sequences generated by RepAHR is higher than that of the repetitive sequences generated by RepARK CLC, RepARK Velvet and REPdenovo.

### Repeats on Repbase sequences

**Fig. 7:**
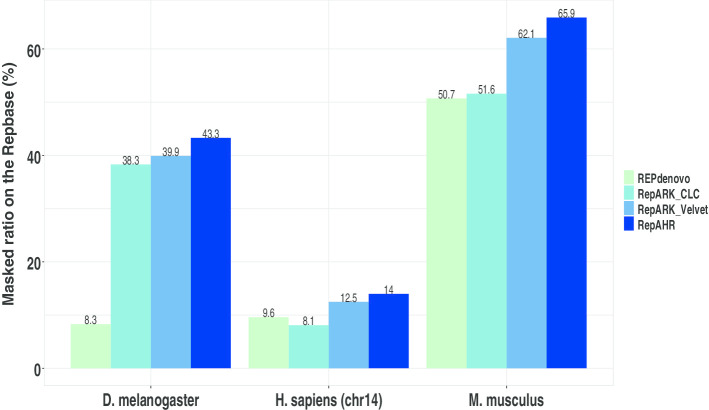
Masked ratios on Repbase sequences. 'Masked ratios on Repbase sequences(%)' is the proportion of bases on the fragments in the repbase library that are covered by the detection results of the four tools

Repbase [22] is the most commonly used database of repetitive DNA sequences. In this study, we use RepeatMakser [21] to compute the ratio of bases in Repbase library that can be covered by detection results of tools. As can be seen from Fig. 7, the masked ratio of RepAHR is higher than that of RepARK CLC, RepARK Velvet and REPdenovo. It is indicated that the repeats identified by RepAHR is closer to the repeats collected in the Repbase database. Therefore, the repeats identified by RepAHR is more accurate and reliable.

BLAST [23] can be used to find the similarity between sequences based on sequence alignment, which is applied to obtain the coverage ratio of the repeats in Repbase that can be covered by the repetitive fragments detected from RepARK CLC, RepARK Velvet, REPdenovo and RepAHR in this study. There are two strategies used to perform the alignment of the repeats identified by each tools with the corresponding Repbase library, which indicate that the fragments in Repbase library can be covered by the single alignment repeats and multiple alignment repeats, respectively. The coverage ratios obtained by using these two strategies are called the best coverage ratio and maximum coverage ratio, which can be abbreviated as BCR and MCR, respectively. Then, we count the number of sequences in Repbase that can be covered by repeats with BCR and MCR from $0\%$ to $100\%$. The number of sequences in Repbase with BCR and MCR more than 50%, 70% and 90% of the four methods is shown in Table 7, respectively. It can be seen that the sequences in Repbase with high BCR and MCR covered by the repeats identified by RepAHR are more than the other three methods.

**Table 7:** The ratios of segments in RepBase library covered by the repetitive sequences generated by each tools (The coverage ratios are calculated by BLAST)

Metrics	Method	> 50%	> 70%	> 90%
BCR	RepARK CLC	87	57	33
	RepARK Velvet	84	42	24
	REPdenovo	19	15	7
	RepAHR	173	116	69
MCR	RepARK CLC	155	115	64
	RepARK Velvet	218	187	149
	REPdenovo	22	19	13
	RepAHR	241	206	161

’BCR’ indicates the best coverage ratio, ’MCR’ indicates the maximum coverage ratio

**Fig. 8:**
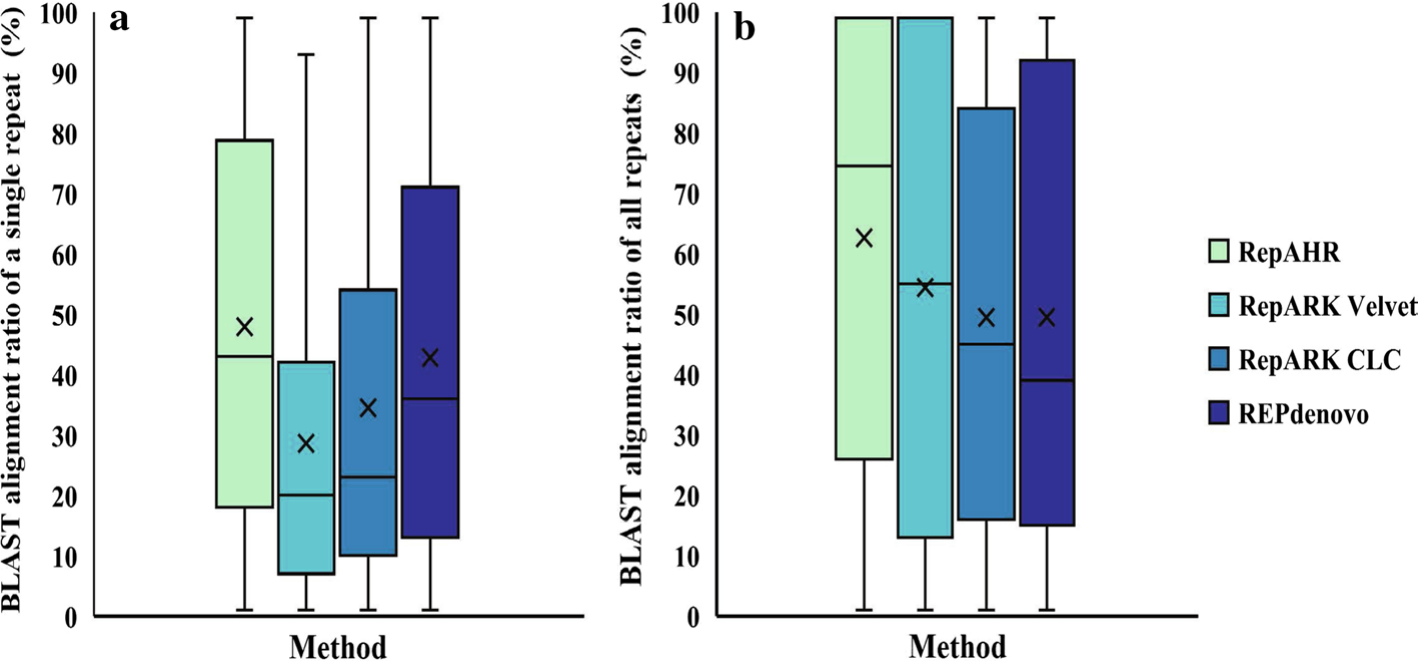
Distribution of BLAST coverage ratios on Repbase sequences. Box plots of BLAST alignment ratios of the repeats identified by the four tools to the repeat segments in the repbase library. Sub-graph **a** shows the case of the distribution of single alignment, and sub-graph **b** shows the case of the distribution of the maximum alignment

Figure 8 shows the distribution of the coverage ratio obtained from the four methods by using the BLAST. Each box shows the range of 50% in the middle, and the horizontal line in the box represents the median of the coverage ratios, the x marked in the box indicates the mean of the coverage ratios. Figure 8a shows the distribution of BCR on the Repbase sequences. As can be seen from this figure,the BCR of the repeats identified by RepAHR is higher than that of the other three methods. This is manifested that the median, mean, and upper quartile and lower quartile of the repeats generated from RepAHR are greater than that of the other three methods. Similarly, the distribution of MCR on the Repbase sequences is shown in Fig. 8b. The upper quartile of the repeats generated from RepAHR is close to 100%, which means that more than 25% of the Repbase sequences are 100% covered by the repeats identified by RepAHR. In addition, the median, mean, and lower quartiles of the repeats generated from RepAHR are higher than those obtained from the other three methods. It is indicated that the repetitive sequences identified by RepAHR are more similar to the repeats in Repbase, and also closer to the standard repetitive sequences.

From the data shown in Tables 5 and 8, we can see that RepAHR has problems with long running time and large memory requirements on some datasets (such as *Acromyrmex echinatior* and *Musmusculus*). Those problems have caused our great concern. To confirm this problem, we retested the CPU time and virtual memory consumption of the three tools on five datasets. Now we summarize the test conclusions as follows: (1) when *RepAHR* uses the default parameters to process some datasets (such as *Acromyrmex echinatior* and *Musmusculus*), it does have the disadvantages of long calculation time and large memory demand. The comparison of CPU time and virtual memory consumption of these three tools when using default parameters to process these five datasets is shown in Table 9; (2) through the analysis, we find that there are two important parameters in *RepAHR* (*MAX_MEMORY* and *THREAD*) that affect the running time and memory consumption. The statistics of the limit effect of the parameter of *MAX_MEMORY* on the memory usage of *RepAHR* when processing the Human-r14 dataset is shown in Table 9. The statistics of the impact of concurrency on the memory usage of *RepAHR* when processing the *Human-r14* dataset is shown in Table 10. As can be seen from the results shown in Table 9 and Table 10, when we lower the values of the two parameters *MAX_MEMORY* and *THREAD*, the memory requirements of the *RepAHR* will be greatly reduced. In addition, the running time of *RepAHR* can also be similar to the other two tools, especially when *MAX_MEMORY* and *THREAD* are set to 20 respectively, just as the shown in Table 11; (3) the causes of the above phenomenon are analyzed. First, the parameter *THREAD* is used to set the number of threads when the program runs in parallel. In theory, the more threads, the shorter computing time, and the higher computing cost. When we reduce the number of threads, we will get the opposite conclusion (the memory cost is reduced, and the running time is longer). However, this statement is only theoretical. In fact, it is not necessary to increase the thread to improve the computing speed. This is because if the number of threads is set too much, it will intensify the competition of system resources and increase the delay of queuing. So in this case, when we appropriately reduce the number of threads, the memory consumption of the algorithm will be reduced on a large scale, and the calculation time of the algorithm will not be greatly affected. Second, the parameter *MAX_MEMORY* is used to set the maximum memory usage of the algorithm. When we reduce the value of this parameter, the operation system will control the amount of memory that the algorithm resides within the set *MAX_MEMORY* range. At this time, the program needs to deal with the limitations of the operation system through page scheduling. Therefore, when we reduce the value of this parameter, the memory consumption of the algorithm will be controlled, but the running speed of the algorithm will also be affected.

**Table 8:** The comparison of CPU time and virtual memory consumption of these three tools when using default parameters to process these five datasets

Species	RepARK [CPU time (s)/virtual memory (kb)]	REPdenovo [CPU time (s)/virtual memory (kb)]	RepAHR [CPU time (s)/virtual memory (kb)]
Drosophila melanogaster	1387.63/9,546,368	4,896.98/13,223,216	4441.25/11,333,456
Saccharomyces cerevisiae	770.96/4,887,024	12,846.08/48,140,200	2,429.36/4,921,672
Acromyrmex echinatior	3198.22/18,730,776	18,747.07/25,592,284	20,245.73/20,513,324
Human chr14	1313.54/4887,452	6,397.10/5,878,436	4,354.44/24,886,024
Musmusculus	9654.50/37,149,572	52,624.49/133,352,464	606,338.15/2,608,150,044

**Table 9:** Statistics of the limit effect of the perameter of *MAX_MEMORY* on the memory usage of *RepAHR* when processing the *Human-r14* dataset

Species	Method	*MAX_MEMORY*	CPU time (s)	Virtual memory (kb)
$Homo \ sapiens$ *chr*14		Default	4354.44	24,886,024
	RepAHR	19	5321.77	4,910,644
		20	5207.86	8,160,520

The parameter *MAX_MEMORY* is used to set the maximum memory usage of the algorithm

**Table 10:** Statistics of the impact of concurrency on the memory usage of *RepAHR* when processing the *Human-r14* dataset

Species	Method	*THREAD*	CPU time (s)	Virtual memory (kb)
$Homo \ sapiens$ *chr*14		10	4880.97	9,552,936
	RepAHR	20	4706.87	9,566,604
		30	4671.14	19,789,184
		40	4354.44	24,886,024

The parameter *THREAD* is used to set the number of threads when the program runs in parallel

**Table 11:** The comparison of CPU time and virtual memory consumption of these three tools when RepAHR using the specific parameters of *MAX_MEMORY=20, THREAD=20* to process these five datasets

Species	RepARK [CPU time (s)/virtual memory (kb)]	REPdenovo [CPU time (s)/virtual memory (kb)]	RepAHR [CPU time (s)/virtual memory (kb)]
Drosophila melanogaster	1387.63/9,546,368	4896.98/13,223,216	4387.63/9,558,308
Saccharomyces cerevisiae	770.96/4,887,024	12,846.08/48,140,200	2378.34/3,911,352
Acromyrmex echinatior	3198.22/18,730,776	18,747.07/25,592,284	19,489.55/18,750,436
Human chr14	1313.54/4,887,452	6397.10/5,878,436	4706.87/9,566,604
Musmusculus	9654.50/37,149,572	52,624.49/133,352,464	7879.77/36,734,056

### Recommendations on the parameters configuration of the proposed tool when resources are limited

Sequence assembly is the most time-consuming and memory-consuming step in the entire processing flow of RepAHR. Therefore, as long as the running time and memory consumption of this step can be controlled by adjusting the parameters *MAX_MEMORY* and *THREADS*, the tool can still run normally under the condition of limited resources. SPAdes uses 512 Mb per thread for buffers, which results in higher memory consumption (The default value of parameters *MAX_MEMORY* and *THREADS* in SPAdes are set to 250GB and 16 respectively). If you set memory limit manually, SPAdes will use smaller buffers and thus less RAM. The parameter *MAX_MEMORY* set memory limit in Gb. SPAdes terminates if it reaches this limit. Actual amount of consumed RAM will be below this limit. Make sure this value is correct for the given machine. SPAdes uses the limit value to automatically determine the sizes of various buffers, etc. The parameter *THREADS* is used to set the number of threads using in SPAdes assembly, and the default value of it is 16. The larger the number of threads is, the faster the SPAdes assembly speed, and the memory consumption will also increase.

Therefore, the value of the parameter *MAX_MEMORY* should be set according to the available memory capacity of the machine. For example, if the available memory of the machine is 300GB, then *MAX_MEMORY* can be set to 250GB at most. It should be noted that SPAdes assembly usually has the minimum memory requirements. For example, when the machine’s available memory are less than 20GB, the tool will report an error. In summary, the value of *MAX_MEMORY* should be taken in the interval [20, available_memory], and the number of threads should be calculated based on the actual processing speed requirements and the overall memory limit.

## Conclusions

In molecular biology, it is important to accurately detect repeats in the DNA sequences. With the development of the next-generation sequencing, more and more tools have been proposed for identification of repeats, including RepARK and REPdenovo. In this paper, we present a new method called RepAHR for de novo repeat identification by assembly of the high-frequency reads. The core steps of RepAHR are as follows: Firstly, RepAHR filters the high-frequency reads from overall NGS data according to certain rules based on the high-frequency *k-mers*. Secondly, it identifies repeats by assembly of the high-frequency reads. The main advantages of RepAHR are reflected in the following two aspects:

1. the high-frequency reads achieve enough coverage and longer than the *k-mers*, which facilitates the assembly process. Compared with the previous two methods, RepAHR not only replaces the high-frequency *k-mers* with the high-frequency reads, but also preserves the information of the paired-end reads as much as possible to assist in the assembly of repetitive sequences. It is well known that the paired-end reads can span hundreds to thousands of bp (base pair), so using its supporting information, RepAHR can assemble and identify longer repetitive regions.
2. due to the sequencing errors and bias, the *k-mers* from the repetitive regions do not necessarily all show high frequencies, and the *k-mers* with low frequencies are not necessarily all from the non-repetitive regions. In addition, in the case of a short *k-mer* size, the error *k-mers* also have the opportunity to couple together and behave as high frequencies. In these cases, it is unreliable to obtain repeating regions directly by assembly of the high-frequency *k-mers*. However, the strategy based on the high-frequency reads assembly can effectively circumvent this problem. Firstly, RepAHR has set a stricter filtering strategy in the process of selecting the high-frequency reads, which makes it less likely that error *k-mers* are used to form repetitive fragments. Secondly, RepAHR also set multiple verification strategies in the process of finalizing the repetitive fragments to ensure that the detection results are authentic and reliable.

The comparison of the repeats identified by RepAHR, RepARK CLC, RepARK Velvet and REPdenovo based on the five NGS datasets. We use multiple metrics, including some basic metrics of the repeats, alignment rate on reference genome, masked ratio on RepBase and so on, to evaluate the performance of each tools. The experimental results show that repeats obtained by RepAHR are more precise and reliable than that of RepARK CLC, RepARK Velvet and REPdenovo.

## Methods

**Fig. 9:**
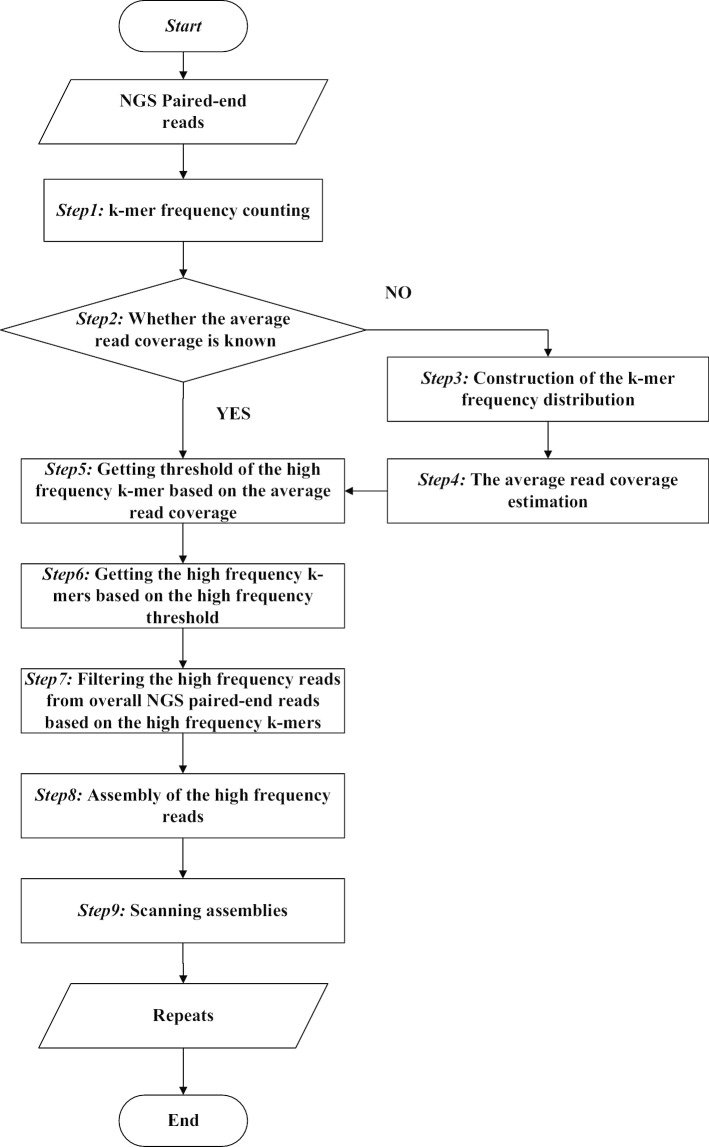
The illustration of the pipeline of RepAHR

As shown in Fig. 9, the proposed method(RepAHR) contains the following phases. Firstly, RepAHR convents overall NGS short paired-end reads into unique *k-mers* and gets their frequencies by using Jellyfish [24]. Secondly, RepAHR needs to determine whether the average read coverage is known. If it is known, its value can be used to calculate the threshold of the high frequency *k-mer* directly. Otherwise, RepAHR needs to estimate it based on the *k-mer* frequency distribution. Thirdly, RepAHR generates a high-frequency *k-mer* set based on overall *k-mers* and the high frequency threshold. Fourthly, RepAHR obtains the high-frequency reads from whole NGS short paired-end reads based on the high-frequency *k-mer* set. Finally, RepAHR gets assemblies of the high-frequency reads, and then scans these contigs to obtain the final repeats.

### Determining the threshold of the high-frequency *k-mer*

#### *k-mer* frequency counting

This section corresponds to *Step1* in the flowchart (Fig. 9). *k-mer* counting can be fulfilled by many available tools, such as Jellyfish [24] and KMC2 [25]. In this step, RepAHR needs to generate two groups of *k-mers* with different lengths by using Jellyfish. The *k-mer* length in the first group is about 15bp–20bp according to the literature [6], while the second group is set to 31 bp which is an empirical value obtained through a lot of experiments.

#### Determining whether the average read coverage is known

This section corresponds to *Step2* in the flowchart (Fig. 9). In this section, RepAHR needs to determine whether the average read coverage is known. If it is known, its value can be used to calculate the threshold of the high frequency *k-mer* directly (the algorithm moves to *Step5*). Otherwise, RepAHR needs to estimate it based on the *k-mer* frequency distribution (the algorithm moves to *Step3*).

#### Constructing the k-mer frequency distribution

This section corresponds to *Step3* in the flowchart (Fig. 9). Suppose *n* *k-mers* and their frequencies are obtained from the statistics in the previous step. Let *K* denote a list containing all *k-mer* and $K_{i}$ be the *i*-th *k-mer* in the list (*i* = 1, 2, 3..., *n*). While list *F* is used to store the frequency of each *k-mer* in *K*, so *F* has a one-to-one relationship with *K*, for example, $F_{i}=t$ indicates that *k-mer* $K_{i}$ appears *t* times in the input reads. Given a frequency number of *t*, the number of *k-mer* that have a frequency number of *t* is denoted as *f*(*t*), and the value of *f*(*t*) can be calculated as follows:

1 $$\begin{aligned} {f(t) = \sum _{i=1}^{|K|}1} \quad if \ {(F_{i}=t)} \end{aligned}$$

**Fig. 10:**
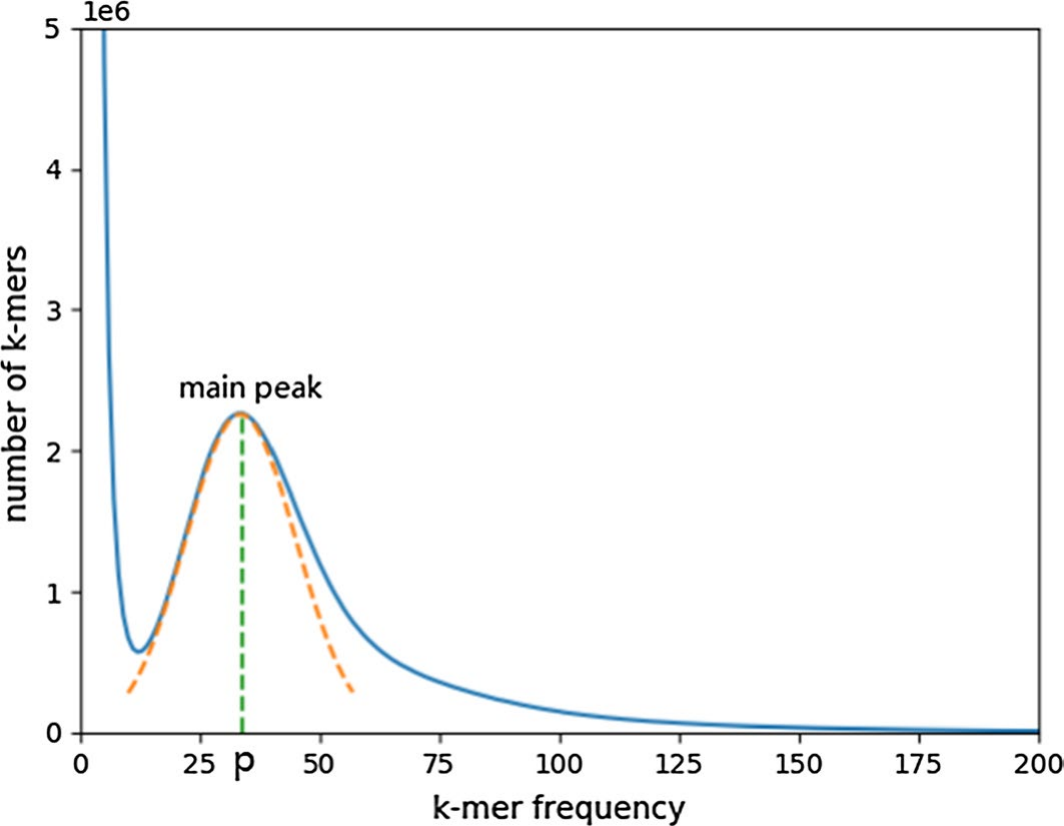
The *k-mer* frequency distribution histogram. In this figure, the blue line is the number of *k-mer* with a specific frequency, the orange dotted line is a Gaussian fit to the trend near the main peak of the blue line, and the green dotted line is the vertical line from the position at the main peak to the x-axis, and *p* is the position where the green dotted line intersects the x-axis

Based on each pair of *t* and *f*(*t*), RepAHR plots the *k-mer* frequency distribution curve as shown in Fig. 10, which is plotted using *Drosophila melanogaster* sequencing data downloaded from the Short Read Archive (http://www.ncbi.nlm.nih.gov/sra), while the x-axis refers to the frequency of *k-mer* and the y-axis refers to the total number of the frequency appearing. If the input read data is evenly distributed over the reference genome, the *k-mer* frequency distribution curve usually forms a Poisson distribution [26] or Gaussian distribution [27] after the steep decreasing at the beginning of the curve, as shown in Fig. 10. According to this feature, we can always find the main peak point of the curve.

#### Estimating the average read coverage

This section corresponds to *Step4* in the flowchart (Fig. 9). RepAHR estimates the average read coverage using a method similar to that in literature [28]. The calculation principle is shown as follows:

2 $$\begin{aligned} {Cov = \frac{p * length}{length - k + 1}} \end{aligned}$$

Where *p* is the horizontal coordinate of the main peak in the *k-mer* frequency distribution histogram, *length* is the average length of the input NGS reads, *k* is the *k-mer* length used in estimation which is settled to 15 by default, and *Cov* is the average read coverage estimated.

#### Getting the threshold of the high-frequency k-mer based on the average read coverage

This section corresponds to *Step5* in the flowchart (Fig. 9). If the average read coverage (*Depth*) is known, RepAHR can multiply it with the coverage factor to obtain the threshold of the high frequency *k-mer* $t_{1}$ ($t_{1}$=*c* $\times$ *Depth* , *c* $\in$ [1.5, 3]) directly. Otherwise, RepAHR needs to multiply the estimated read coverage *Cov* generated in the previous step by the coverage factor *c* to obtain the threshold $t_{1}$ ($t_{1}$=*c* $\times$ *Cov* , *c* $\in$ [1.5, 3]). Where *c* is reasonable to set between 1.5 and 3, the larger *c* is, the more stringent the selection of the high-frequency *k-mer* is.

### Getting the high-frequency k-mers based on the high frequency threshold

This section corresponds to *Step6* in the flowchart (Fig. 9). In this section, RepAHR first obtains a *k-mer* set with a length of about 31 bp generated in the *Step1*. After that, the $k-mer _{i}$ whose frequency *f*(*i*) is lower than the threshold $t_{1}$ are discarded, and the remaining *k-mers* are composed as a high-frequency *k-mer* set as $S_{h}$. The principle of generating $S_{h}$ is shown as follows.

3 $$\begin{aligned} S_{h}=\{k-mer _{i}| \forall k-mer _{i} \in K, f(i) \ge t_{1} \} \end{aligned}$$

### Filtering the high frequency reads from overall NGS reads based on the high frequency k-mers

**Fig. 11:**
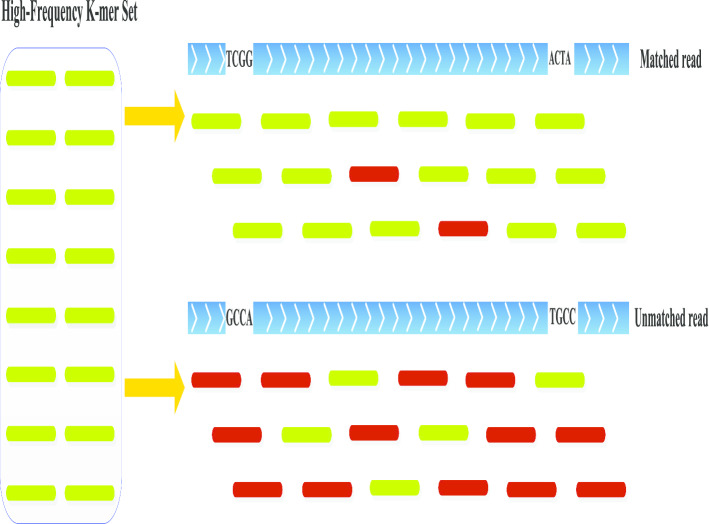
Schematic diagram of generating the high-frequency reads. In this figure, a green line on the left denotes a high-frequency *k-mer*, all these *k-mers* constitute a high-frequency *k-mer* set. The blue line denotes the NGS reads, and the green and red line segments under the blue line represent all the *k-mers* generated from an NGS read. A green line denotes a *k-mer* which appears in the high-frequency *k-mer* set, and a red line denotes a *k-mer* which does not appear in the high-frequency *k-mer* set. The diagram contains a matched case and an unmatched case on the right

This section corresponds to *Step7* in the flowchart (Fig. 9). For each read, all *k-mers* included in them can be denoted as *S*={$s_{1}$, $s_{2}$, ..., $s_{q}$}, respectively. RepAHR checks how many *k-mers* in *S* appear in the high frequency *k-mer* set $S_{h}$. If both the *k-mers* at the beginning and at the end of a read ($s_{1}$ and $s_{q}$) are included in set $S_{h}$, and the number of the high-frequency *k-mer* in *S* has reach a certain percentage threshold $t_{2}$. Then the read is classified as a high-frequency read. RepAHR sets $t_{2}$ to 90% by default. The principle of this process is shown in Fig. 11.

### Assembling the high frequency reads

This section corresponds to *Step8* in the flowchart (Fig. 9). In this phase, SPAdes [14] is used to achieve the contigs of the high-frequency reads. Subsequently, the contigs with low coverage are filtered out [29]. The contigs left are the initial repeats determined by RepAHR.

### Scanning assemblies

This section corresponds to *Step9* in the flowchart (Fig. 9). In this step, RepAHR needs to sort the initial repetitive sequences generated from SPAdes according to the fragment length, and removes some fragments whose length cannot meet the requirements. After that, the fragments left are the final repeats determined by RepAHR.

### Experimental datasets

In this study, five data sets are used to compare the repeats identified by RepAHR, RepARK CLC, RepARK Velvet and REPdenovo. Five species are *Drosophila melanogaster*, *Saccharomyces cerevisiae*, *Acromyrmex echinatior*, *Mus musculus* and *Homo sapiens Chromosome 14*. Five data sets are downloaded from NCBI SRA database (https://www.ncbi.nlm.nih.gov/sra/) and GAGE website (http://gage.cbcb.umd.edu/) [30] . More information about the datasets is shown in the Table 12.

**Table 12:** Information about the NGS datasets

Species	Dataset source	Dataset name	Dataset size (GB)
$Drosophila\ melanogaster$	NCBI SRA	SRX040484 SRX040486	6.3
$Saccharomyces\ cerevisiae$	NCBI SRA	SRR6846984	1.7
$Acromyrmex\ echinatior$	NCBI SRA	ERR034186	10.7
$Mus\ musculus$	NCBI SRA	ERR2894257 ERR2894259 ERR2894260	55.6
$Homo\ sapiens\ chr14$	GAGE	DataSet3 Library 1	9.8

## Data Availability

The tool is publicly available at https://github.com/bioinfomaticsCSU/RepAHR.

## Abbreviations

NGS: next generation sequencing;
TEs: transposable elements;
TRs: tandem repeats;
BCR: the best coverage ratio;
MCR: the maximum coverage ratio.

## References

[1] Janicki M, Rooke R, Yang G (2011). Bioinformatics and genomic analysis of transposable elements in eukaryotic genomes. Chromosome Res.

[2] de Koning AJ, Gu W, Castoe TA, Batzer MA, Pollock DD (2011). Repetitive elements may comprise over two-thirds of the human genome. PLoS Genet.

[3] Ouyang S, Buell CR (2004). The TIGR plant repeat databases: a collective resource for the identification of repetitive sequences in plants. Nucleic Acids Res.

[4] Castro JP, Carareto CM (2004). Drosophila melanogaster P transposable elements: mechanisms of transposition and regulation. Genetica.

[5] Treangen TJ, Salzberg SL (2012). Repetitive DNA and next-generation sequencing: computational challenges and solutions. Nat Rev Genet.

[6] Kurtz S, Narechania A, Stein JC, Ware D (2008). A new method to compute K-mer frequencies and its application to annotate large repetitive plant genomes. BMC Genomics.

[7] Novák P, Neumann P, Pech J, Steinhaisl J, Macas J (2013). RepeatExplorer: a Galaxy-based web server for genome-wide characterization of eukaryotic repetitive elements from next-generation sequence reads. Bioinformatics.

[8] Koch P, Platzer M, Downie BR (2014). RepARK-de novo creation of repeat libraries from whole-genome NGS reads. Nucleic Acids Res.

[9] Fertin G, Jean G, Radulescu A, Rusu I (2015). Hybrid de novo tandem repeat detection using short and long reads. BMC Med Genomics.

[10] Guo R, Li Y-R, He S, Ou-Yang L, Sun Y, Zhu Z (2017). RepLong: de novo repeat identification using long read sequencing data. Bioinformatics.

[11] Chu C, Nielsen R, Wu Y (2016). REPdenovo: inferring de novo repeat motifs from short sequence reads. PLoS ONE.

[12] Luo J, Wang J, Zhang Z, Wu F-X, Li M, Pan Y (2014). EPGA: de novo assembly using the distributions of reads and insert size. Bioinformatics.

[13] Luo J, Wang J, Li W, Zhang Z, Wu F-X, Li M, Pan Y (2015). EPGA2: memory-efficient de novo assembler. Bioinformatics.

[14] Bankevich A, Nurk S, Antipov D, Gurevich AA, Dvorkin M, Kulikov AS, Lesin VM, Nikolenko SI, Pham S, Prjibelski AD (2012). SPAdes: a new genome assembly algorithm and its applications to single-cell sequencing. J Comput Biol.

[15] Liao X, Li M, Zou Y, Wu F, Pan Y, Wang J (2019). Current challenges and solutions of de novo assembly. Quant Biol.

[16] Liao X, Zhang X, Wu F, Wang J. de novo repeat detection based on the third generation sequencing reads. In: 2019 IEEE international conference on bioinformatics and biomedicine (2019BIBM). 10.1109/BIBM47256.2019.8982959.

[17] Li M, Liao Z, He Y, Wang J, Luo J, Pan Y (2017). ISEA: iterative seed-extension algorithm for de novo assembly using paired-end information and insert size distribution. IEEE/ACM Trans Comput Biol Bioinform: TCBB.

[18] Robinson JT, Thorvaldsdóttir H, Wenger AM, Zehir A, Mesirov JP (2017). Variant review with the integrative genomics viewer. Cancer Res.

[19] Liao X, Li M, Zou Y, Wu F, Pan Y, Luo F, Wang J (2019). EPGA-SC: a framework for de novo assembly of single-cell sequencing reads. IEEE/ACM Trans Comput Biol Bioinform.

[20] Langmead B, Salzberg SL (2012). Fast gapped-read alignment with Bowtie 2. Nat Methods.

[21] Chen N (2004). Using RepeatMasker to identify repetitive elements in genomic sequences. Curr Protoc Bioinform.

[22] Jurka J, Kapitonov VV, Pavlicek A, Klonowski P, Kohany O, Walichiewicz J (2005). Repbase update, a database of eukaryotic repetitive elements. Cytogenet Genome Res.

[23] Altschul SF, Gish W, Miller W, Myers EW, Lipman DJ (1990). Basic local alignment search tool. J Mol Biol.

[24] Marçais G, Kingsford C (2011). A fast, lock-free approach for efficient parallel counting of occurrences of k-mers. Bioinformatics.

[25] Deorowicz S, Kokot M, Grabowski S, Debudaj-Grabysz A (2015). KMC 2: fast and resource-frugal k-mer counting. Bioinformatics.

[26] Li X, Waterman MS (2003). Estimating the repeat structure and length of DNA sequences using l-tuples. Genome Res.

[27] Kelley DR, Schatz MC, Salzberg SL (2010). Quake: quality-aware detection and correction of sequencing errors. Genome Biol.

[28] Liao X, Li M, Zou Y, Wu F, Pan Y, Luo F, Wang J (2018). Improving de novo assembly based on read classification. IEEE/ACM Trans Comput Biol Bioinform.

[29] Liao X, Li M, Zou Y, Wu F, Pan Y, Wang J (2019). An efficient trimming algorithm based on multi-feature fusion scoring model for NGS data. IEEE/ACM Trans Comput Biol Bioinform.

[30] Wu B, Li M, Liao X, Luo J, Wu F, Pan Y, Wang J (2018). MEC: misassembly error correction in contigs based on distribution of paired-end reads and statistics of gc-contents. IEEE/ACM Trans Comput Biol Bioinform.

